# Polarization-resolved second-harmonic-generation imaging of dermal collagen fiber in prewrinkled and wrinkled skins of ultraviolet-B-exposed mouse

**DOI:** 10.1117/1.JBO.24.3.031006

**Published:** 2018-09-06

**Authors:** Shu-ichiro Fukushima, Makoto Yonetsu, Takeshi Yasui

**Affiliations:** a Osaka University, Graduate School of Engineering Science, Toyonaka, Japan; b Tokushima University, Graduate School of Technology, Industrial and Social Sciences, Tokushima, Japan

**Keywords:** skin, photoaging, collagen, wrinkle, orientation, second-harmonic-generation microscopy

## Abstract

Skin wrinkling is a typical symptom of cutaneous photoaging; however, the skin wrinkling depends on not only the actual age but also exposure history to ultraviolet B (UVB) rays in individuals. Therefore, there is considerable need for its assessment technique *in vivo* in skin cosmetics and antiaging dermatology. Wrinkles always appear as linear grooves in the skin, and dermal collagen fibers play an important role to determine the morphology and mechanical properties of the skin. Therefore, an optical probe sensitive to dermal collagen fiber and its orientation will be useful. Polarization-resolved second-harmonic-generation (SHG) microscopy is a promising approach for *in vivo* evaluation of collagen fiber orientation because the efficiency of SHG light is sensitive to collagen fiber orientation when the incident light is linearly polarized. We investigate orientation change of dermal collagen fiber in prewrinkled and wrinkled skins of the UVB-exposed mouse model using polarization-resolved SHG microscopy. A polarization anisotropic image of the SHG light indicates that the change of collagen fiber orientation starts in the prewrinkled skin of UVB-exposed mice, then the wrinkle appears. Furthermore, the dominant direction of collagen fiber orientation in the prewrinkled skin is significantly parallel to the wrinkle direction in the wrinkled skin. This result implies that the change of collagen fiber orientation is a trigger of wrinkling in cutaneous photoaging.

## 1 Introduction

Cutaneous aging is an age-related skin change and is closely related to a balance change of production and decomposition of dermal collagen fibers. The cutaneous aging is classified into intrinsic aging and extrinsic aging. The intrinsic aging, also known as chronological aging, is caused by the fact that the fibroblast decreases its number and activity to produce the dermal collagen fibers as one grows older. In the other words, the intrinsic aging is a physiological phenomenon that no one can avoid and is correlated with the actual age. On the other hand, extrinsic aging is skin change or damage caused by extrinsic factors. For example, exposure of the skin to ultraviolet B (UVB) rays in sunlight often accelerates skin aging and causes morphological changes in the skin, such as mottled pigmentation, leathery texture, laxity, sallowness, and deep wrinkle. This is called cutaneous photoaging.^1^ In the cutaneous photoaging, decomposition of dermal collagen fibers is more dominant than production of them because the UVB-damaged collagen fibers are decomposed by active secretion of collagenase. In contrast to the intrinsic aging, the photoaging depends on not only the actual age but also history of UVB exposure in individuals. Therefore, the need exists for an *in vivo* assessment technique to estimate the degree of cutaneous photoaging for studies in fields such as skin cosmetics and antiaging dermatology.

Cutaneous photoaging is closely related to the quantity and structure of collagen fibers in the superficial dermis because dermal collagen fibers contribute to the morphology and mechanical properties of the skin. Second-harmonic-generation (SHG) microscopy^2^ is an attractive tool for *in vivo* monitoring of dermal collagen fiber due to unique imaging characteristics, such as high image contrast, high spatial resolution, optical three-dimensional (3-D) sectioning, low invasiveness, deep penetration, no interference from background light, and most importantly selective visualization *in vivo* of collagen fibers without additional staining. One promising approach for assessment of cutaneous photoaging is a combination of SHG microscopy with two-photon excited autofluorescence microscopy because it gives information on an abundance ratio of collagen fibers and elastin fibers in the dermis.^3^ Such an approach was effectively used for *in vivo* assessment of human skin aging^4^ and collagen remodeling after microablative fractional laser resurfacing.^5^ Another promising approach is the use of SHG image analysis.^6^^,^^7^ For example, the texture analysis of SHG images was applied for quantitative analysis of reticular dermal collagen fiber *in vivo* in human facial cheek skin and indicated a significantly higher correlation with skin elasticity measured by a Cutometer^®^.^8^

From the viewpoint of assessment of deep wrinkles characteristic of UVB-exposed skin, polarization-resolved SHG microscopy can provide interesting insights regarding the direction of collagen fibers, i.e., collagen fiber orientation.^9^^–^^13^ As the efficiency of SHG light is sensitive to collagen fiber orientation when the incident light is linearly polarized, polarization-resolved SHG microscopy is effective for investigating collagen fiber orientation in the dermis. Previously, we reported that wrinkle direction in UVB-exposed mice skin is significantly parallel to the orientation of dermal collagen fibers.^14^ However, it is not clear which came first, the wrinkle or the collagen fiber orientation change.

In this paper, we investigate orientation change of dermal collagen fiber in prewrinkled and wrinkled skins of UVB-exposed mice using polarization-resolved SHG microscopy.

## 2 Materials and Methods

### 2.1 Sample

**Fig. 1:**
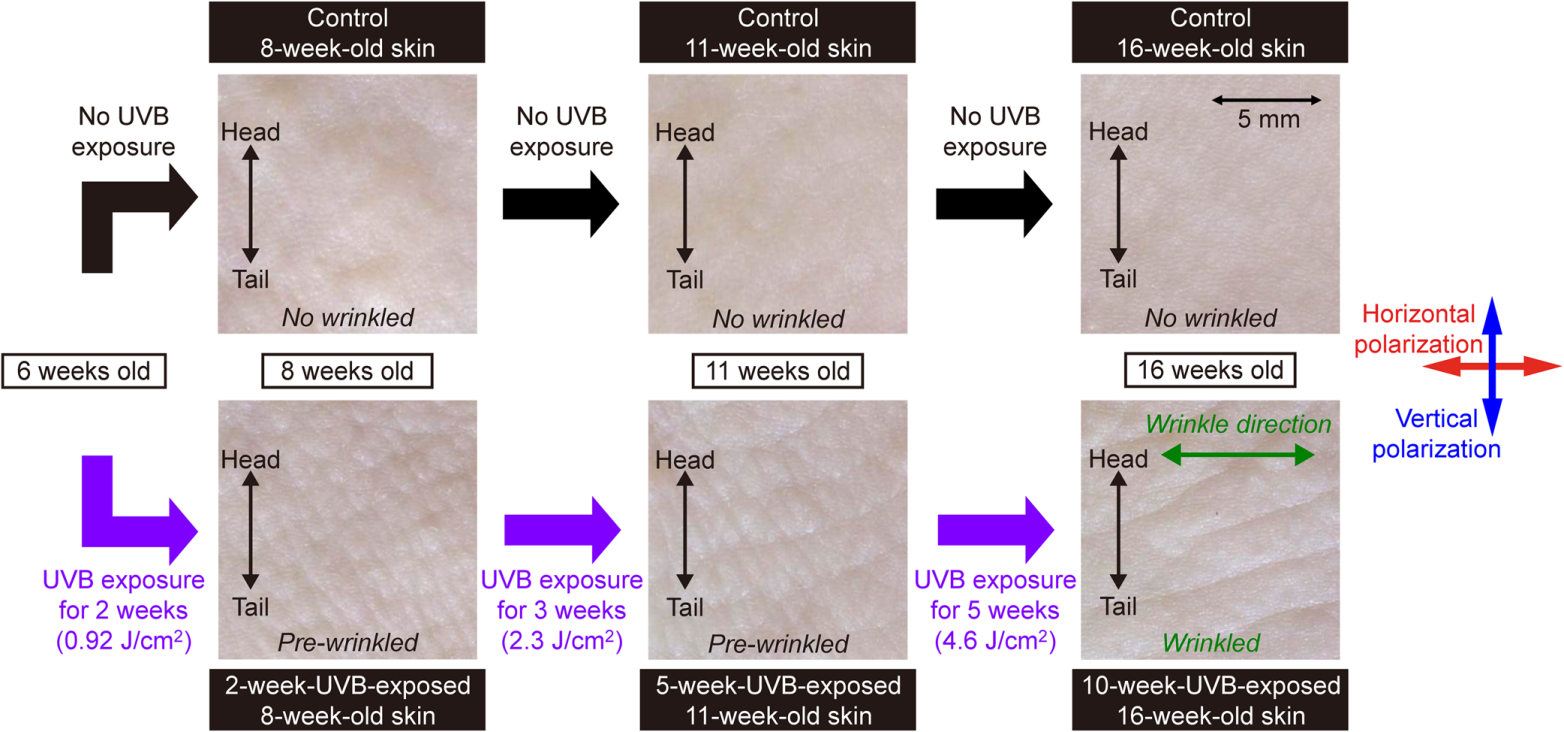
Protocol of sample preparation and optical photographs of skin surface of 2-week-UVB-exposed (8 weeks old), 5-week-UVB-exposed (11 weeks old), 10-week-UVB-exposed (16 weeks old), and three age-matched control samples (8, 11, and 16 weeks old). Deep wrinkles were observed in the 10-week-UVB-exposed skin. Vertical polarization of the laser light is parallel to the meridian line of the body while UVB-induced wrinkles run along the horizontal polarization of it.

UVB-exposed albino hairless mice (HR-1) were selected as a model of cutaneous photoaging. Wrinkles were induced by repetitive low-dose UVB irradiation to the back of the hairless mice according to the method used by Kligman^15^ with slight modification. In our previous paper, when the mice skin was exposed intermittently to low-dose UVB light for 10 weeks (total UVB exposure dose $\approx 4.6{\;J/\mathrm{cm}}^{2}$), deep wrinkles appeared in the mouse back perpendicular to the meridian line of the body in 16-week-old mice.^14^ We then selected exposure periods of low-dose UVB light of 2 and 5 weeks to prepare prewrinkled skin, respectively. Figure 1 shows the sample preparation protocol and optical photographs of the corresponding skin samples. For prewrinkled and wrinkled skins, we used three kinds of UVB-exposed models of albino hairless mice: (1) 2-week-UVB-exposed skin, which was exposed intermittently to low-dose UVB light from 6 to 8 weeks of age (8 weeks old, total UVB exposure dose $\approx 0.92\;{J/\mathrm{cm}}^{2}$), (2) 5-week-UVB-exposed skin, which was exposed intermittently to low-dose UVB light from 6 to 11 weeks of age (11 weeks old, total UVB exposure dose $\approx 2.3\;{J/\mathrm{cm}}^{2}$), and (3) 10-week-UVB-exposed skin, which was exposed intermittently to low-dose UVB light from 6 to 16 weeks of age (16 weeks old, total UVB exposure dose $\approx 4.6\;{J/\mathrm{cm}}^{2}$) as shown in a lower panel of Fig. 1. In the 2-week-UVB-exposed 8-week-old skin and 5-week-UVB-exposed 11-week-old skin, wrinkles did not appear although a somewhat rough skin surface was observed (namely, prewrinkled skin). However, deep wrinkles appeared on the mouse back perpendicular to the meridian line of the body in 16-week-old mice (namely, wrinkled skin), which is similar to our previous paper.^14^ The 8-week-old skin, 11-week-old skin, and 16-week-old skin without any UVB irradiation were used as age-matched control, showing no wrinkles (see an upper panel of Fig. 1). UVB-exposed and age-matched control skin samples were excised from the back of the hairless mice. The number of samples was 8 for each group.

### 2.2 Experimental Setup

**Fig. 2:**
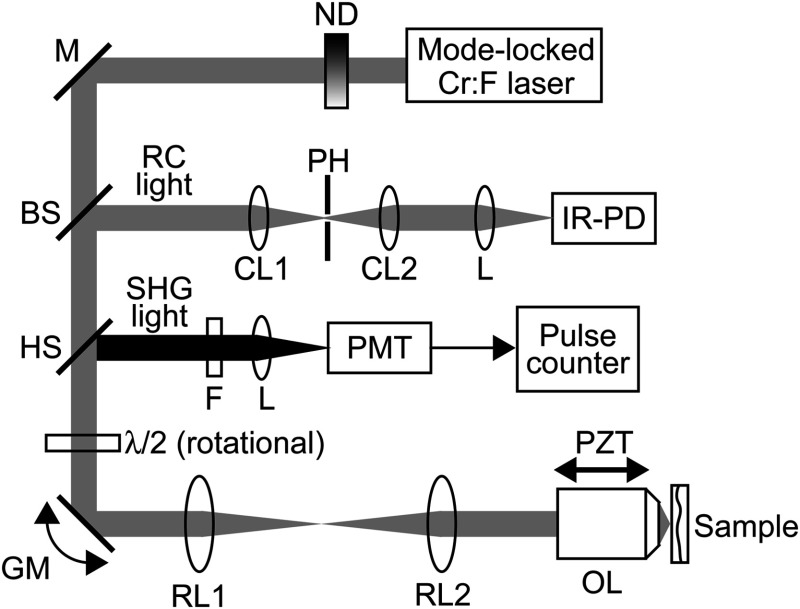
Experimental setup. ND, neutral density filter; M, mirror; HS, harmonic separator; λ/2, half waveplate; GM, galvano mirrors; RL1 and RL2, relay lenses; OL, objective lens; PZT, piezoelectric transducer; F, infrared-cut filter; Ls, lenses; PMT, photon-counting-type photomultiplier; RC light, reflectance fundamental light; BS, beam splitter; CL1 and CL2, confocal lenses; PH, 50-μm diameter pinhole; IR-PD, infrared photodetector.

Figure 2 shows an experimental setup of the polarization-resolved SHG microscopy. A 1250-nm mode-locked Cr:Forsterite laser pumped by a 7.5 W Ytterbium fiber laser running at 1064 nm (Avesta Project Ltd., CrF-65P) was used for this study. The laser pulse has a duration of 90 fs and an average power of 200 mW at a repetition rate of 73 MHz. For rapid acquisition of SHG images, the laser beam was scanned two-dimensionally by a pair of galvano mirrors (GM). After passing through relay lenses (RL1 and RL2), the laser beam was focused onto the sample with an objective lens (OL; Nikon Instruments Inc. CFI Plan 50×H, magnification=50, NA=0.9, WD=350  μm, oil-immersion). The OL can be moved along the optical axis by a piezoelectric transducer (PZT, stroke length=350  μm). Combination of the GM with the PZT enables optical 3-D sectioning images of SHG light. Average power of the laser light on the sample was set to be 42 mW by a neutral density filter (ND). A portion of the generated SHG light was backscattered into the sample and then was collected via the OL. The SHG light was descanned by the GM and separated from the laser light by a harmonic separator (HS, reflected wavelength=625  nm) and an infrared-cut filter (F, stop wavelength>800  nm). The SHG light was detected by the combination of a photon-counting-type photomultiplier (PMT, Hamamatsu Photonics K. K., H8259-01) and a pulse counter. For the polarization measurement of the SHG light, a half waveplate (λ/2) for 1250 nm was inserted in the optical path and was used for polarization control of the laser light. As this λ/2 acted as a full waveplate to the 625-nm SHG light, the polarization angle of the SHG light did not change by passing through the λ/2 in the return SHG path. HS has a polarization dependence of its reflectivity and its transmission,^16^ and the scanning GM has a dependence of its reflectivity on the scanning angle. To cancel these influences, we compensated the polarization-resolved SHG images of skin samples by measuring a micropowder of a nonlinear optical crystal as an isotropic SHG sample and using its SHG image as a reference.

To visualize overall tissue structure of the skin and determine the depth from the skin surface, a confocal imaging setup of the reflectance fundamental light (RC light) also was added to the microscope. The reflectance fundamental light from the sample passed through the HS and was partly reflected by a beam splitter (BS). A confocal section was composed of a pair of lens with a focal length of 50 mm (CL1 and CL2) and a 50-μm diameter pinhole (PH). The fundamental light passing through the confocal setup then was detected by an infrared photodetector (IR-PD).

## 3 Results

**Fig. 3:**
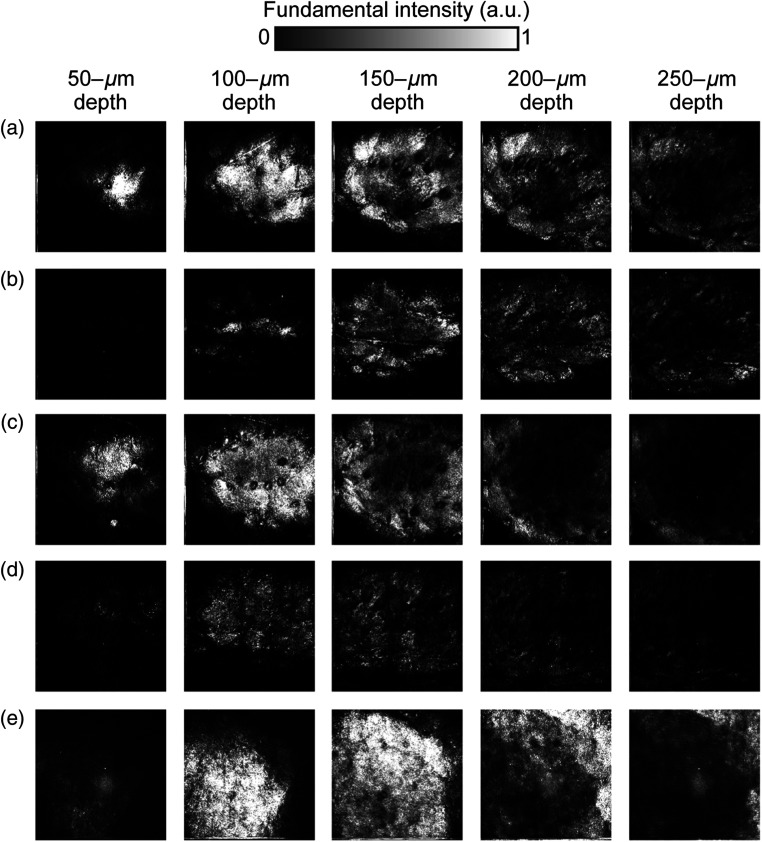
Comparison of depth-resolved confocal images. (a) 2-week-UVB-exposed 8-week-old skin, (b) control 8-week-old skin, (c) 5-week-UVB-exposed 11-week-old skin, and (d) control 11-week-old skin, and (e) 10-week-UVB-exposed 16-week-old skin. Image size=800  μm×800  μm, corresponding to 256  pixels×256  pixels.

First, we measured the confocal images of the UVB-exposed and age-matched control skin samples. Figure 3 shows depth-resolved confocal images of dermal tissue at a step of 50  μm: (a) 2-week-UVB-exposed 8-week-old skin, (b) control 8-week-old skin, (c) 5-week-UVB-exposed 11-week-old skin, (d) control 11-week-old skin, and (e) 10-week-UVB-exposed 16-week-old skin. The area of each image is 800  μm×800  μm, composed of 256  pixels×256  pixels, with an image acquisition time of 10  s/image. The skin surface was defined as 0  μm in depth. The image contrast is given by scattering in tissue: mainly melanin pigment deposited on a boundary between the epidermis layer and the dermis layer. However, there are no significant differences among different skin samples although the internal structure of the skin changes depending on the probing depth.

**Fig. 4:**
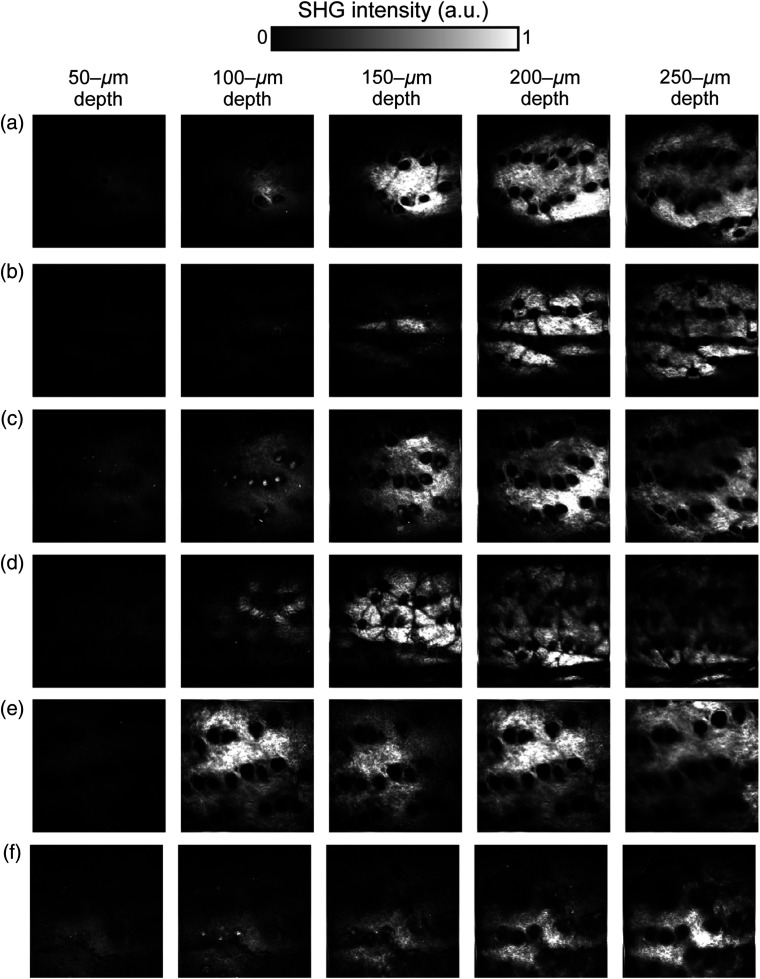
Comparison of depth-resolved SHG images. (a) 2-week-UVB-exposed 8-week-old skin, (b) control 8-week-old skin, (c) 5-week-UVB-exposed 11-week-old skin, and (d) control 11-week-old skin, (e) 10-week-UVB-exposed 16-week-old skin, and (f) control 16-week-old skin. Image size=800  μm×800  μm, corresponding to 256  pixels×256  pixels.

Second, the depth-resolved SHG image of the UVB-exposed and age-matched control skin samples were measured. The incident laser light was linearly polarized in the vertical direction, which is parallel to the meridian line of the body. Figure 4 shows SHG image of dermal collagen fibers at a step of 50  μm: (a) 2-week-UVB-exposed 8-week-old skin, (b) control 8-week-old skin, (c) 5-week-UVB-exposed 11-week-old skin, (d) control 11-week-old skin, (e) 10-week-UVB-exposed 16-week-old skin, and (f) control 16-week-old skin (image size=800  μm×800  μm, pixel size=256  pixels×256  pixels, and image acquisition time=10  s/image). Due to the different contrast mechanism from the confocal image, the SHG image selectively visualizes spatial distribution of the dermal collagen fibers. In Fig. 4, while the meridian line of the sample is in the vertical direction, the deep wrinkles in the 10-week-UVB-exposed 16-week-old skin run along the horizontal direction. Hair follicles appeared as black holes and were aligned perpendicular to the meridian line. However, no significant difference in the structure of dermal collagen fibers among different skin samples was observed.

**Fig. 5:**
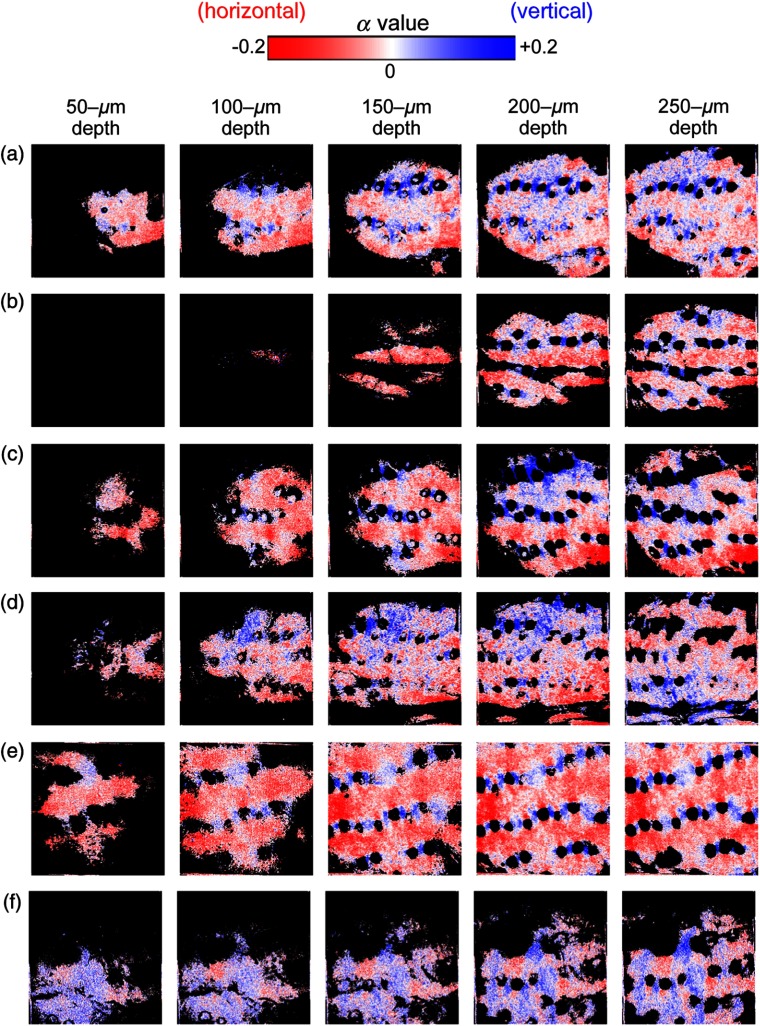
Comparison of depth-resolved α images. (a) 2-week-UVB-exposed 8-week-old skin, (b) control 8-week-old skin, (c) 5-week-UVB-exposed 11-week-old skin, and (d) control 11-week-old skin, (e) 10-week-UVB-exposed 16-week-old skin, and (f) control 16-week-old skin. Image size=800  μm×800  μm, corresponding to 256  pixels×256  pixels.

To obtain a detailed distribution of collagen fiber orientation in the dermis, polarization-resolved SHG imaging was applied for the samples. To investigate the collagen fiber orientation quantitatively, polarization anisotropy of SHG light (α) was defined using the following equation:^13^^,^^14^

$$\alpha =\frac{I_{v}-I_{H}}{I_{v}+I_{H}},$$ (1)

where $I_{V}$ and $I_{H}$ are SHG intensities when the incident light is vertically (parallel to the meridian line) and horizontally (perpendicular to it) polarized, respectively. Then, the α image was calculated by substituting $I_{V}$ and $I_{H}$ values at each pixel of two vertically and horizontally polarization-resolved SHG images for Eq. (1). The resulting α images are shown in Fig. 5: (a) 2-week-UVB-exposed 8-week-old skin, (b) control 8-week-old skin, (c) 5-week-UVB-exposed 11-week-old skin, (d) control 11-week-old skin, (e) 10-week-UVB-exposed 16-week-old skin, and (f) control 16-week-old skin. They reflect the distribution of collagen fiber orientation. Collagen fiber orientation is uniaxial for α=±1 and random or biaxial for α=0. The sign of α value provides the dominant direction of the collagen fiber orientation: positive for a vertical orientation (blue) and negative for a horizontal orientation (red). The most noticeable difference was confirmed between the 10-week-UVB-exposed skin with deep wrinkles [see Fig. 5(e)] and the age-matched control skin [see Fig. 5(f)]; reddish α image for the wrinkled skin and bluish α image for the control skin. This feature was in good agreement with that in our previous paper.^14^ Such difference was confirmed on 2-week-UVB-exposed skin and 5-week-UVB-exposed skin without wrinkles; however, it becomes smaller than the 10-week-UVB-exposed skin.

**Fig. 6:**
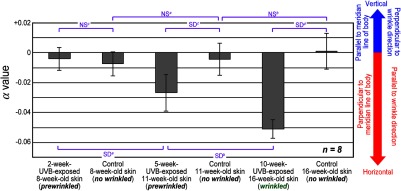
Statistics of mean values in α image for six groups of skin sample (n=8 for each group). SD, significant different (P<0.05). NS, not significant.

To confirm the significant difference of α values, we calculated the mean and standard deviation of α values in the α image at the depth that SHG image indicates the highest intensity. Figure 6 shows the statistics of the α values for 2-week-UVB-exposed, 5-week-UVB-exposed, and 10-week-UVB-exposed skin together with three age-matched control skins, indicating the significant difference among them (n=8, P<0.05). First, different groups of age-matched control skin (control 8-week-old skin, control 11-week-old skin, and control 16-week-old skin) were not significant (see ${\mathrm{NS}}^{a}$ and ${\mathrm{NS}}^{b}$ in Fig. 6). Second, different groups of UVB-exposed skin (2-week-UVB-exposed 8-week-old skin, 5-week-UVB-exposed 11-week-old skin, and 10-week-UVB-exposed 16-week-old skin) have significant difference (see ${\mathrm{SD}}^{a}$ and ${\mathrm{SD}}^{b}$ in Fig. 6). Third, when comparing UVB-exposed skin and age-matched control skin, significant difference was confirmed between 5-week-UVB-exposed skin and age-matched control skin (see ${\mathrm{SD}}^{c}$ in Fig. 6), and also 10-week-UVB-exposed skin and age-matched control skin (see ${\mathrm{SD}}^{d}$ in Fig. 6); however, there are no significant differences between 2-week-UVB-exposed skin and age-matched control skin (see ${\mathrm{NS}}^{c}$ in Fig. 6).

## 4 Discussion

We first discuss findings deduced from the statistical analysis in Fig. 6. From ${\mathrm{NS}}^{a}$ and ${\mathrm{NS}}^{b}$ in Fig. 6, we can ignore the influence of intrinsic aging or chronological aging in the skin of the present animal model because the age-dependent change of the α value was not confirmed; we can focus on only the influence of UVB irradiation on the skin in this model. ${\mathrm{SD}}^{a}$ and ${\mathrm{SD}}^{b}$ in Fig. 6 indicated that the α value changes toward the negative value depending on the total dose of UVB exposure. It is important to note that the 2-week-UVB-exposed 8-week-old skin and the 5-week-UVB-exposed 11-week-old skin were prewrinkled with no wrinkles, whereas the 10-week-UVB-exposed 16-week-old skin was wrinkled with deep wrinkles along the horizontal direction (see Fig. 1). In the other words, UVB exposure alters the orientation of dermal collagen fibers toward the direction perpendicular to the meridian line of the body; this direction is significantly parallel to the running direction of skin wrinkles observed in the 10-week-UVB-exposed 16-week-old skin. ${\mathrm{NS}}^{c}$ in Fig. 6 shows that the UVB-exposure-dependent change of collagen fiber orientation is still too small; however, such UVB-exposure-dependent change significantly occurs in 5-week-UVB-exposed 11-week-old skin and 10-week-UVB-exposed 16-week-old skin from ${\mathrm{SD}}^{c}$ and ${\mathrm{SD}}^{d}$ in Fig. 6, respectively. From these findings, we can conclude that the collagen fiber orientation change comes first, then the wrinkle appears.

We next consider the mechanism of the skin wrinkle formation. First, collagen fiber has higher mechanical strength along its axial direction but lower one across it. Second, usual skin has neutral or random orientation of collagen fibers as shown in the age-matched control skin of Fig. 6. Third, the wrinkles always appear as linear grooves in the skin. On the other hand, it is considered that portions of skin fall as a result of the lost tension in the skin so that the wrinkles are formed. In the 2-week-UVB-exposed 8-week-old skin and the 5-week-UVB-exposed 11-week-old skin, the wrinkle did not appear due to smaller anisotropy of collagen fiber orientation. However, if the anisotropy of collagen fiber orientation becomes larger like the 10-week-UVB-exposed 16-week-old skin, the skin wrinkle appears along the direction of the collagen fiber orientation due to difference of the mechanical strength between parallel and orthogonal to the collagen fiber axis.

**Fig. 7:**
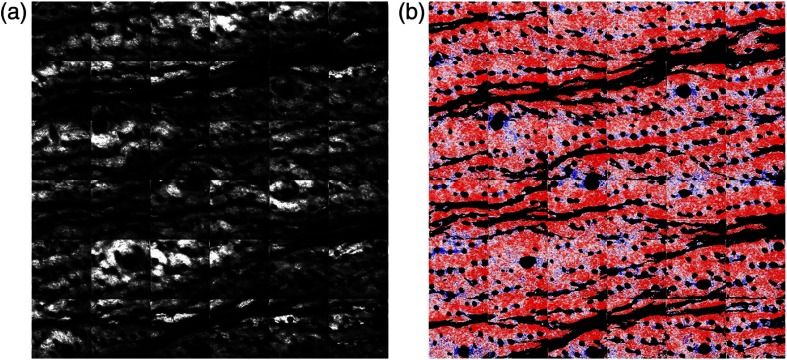
Large-area (a) SHG image and (b) α image of the wrinkles in 10-week-UVB-exposed 16-week-old skin. Image size=3.6  mm×3.6  mm, corresponding to 1536  pixels×1536  pixels.

We confirmed a close relationship between the wrinkle direction and the collagen fiber orientation in the prewrinkled and wrinkled skins from Fig. 6. However, the α image of the wrinkled skin [see Fig. 5(e)] could not capture the skin winkle itself due to the limitation of its field of view. It is interesting to visualize the α image of the skin wrinkle and compare it with that of its surrounding skin. To this end, we expanded the field of view in the SHG image up to 3.6  mm×3.6  mm. By scanning GM, we acquired an SHG image of 0.6-mm×0.6-mm region, composed of 256  pixels×256  pixels. To enlarge the imaging region, we laterally scanned the sample position at intervals of 0.6 mm using a stepping-motor-driven translation stage whenever acquiring an SHG image of 0.6-mm×0.6-mm regions using GM. Finally, a large-area SHG image was obtained by stitching together 36 SHG images in a matrix of six rows and six columns. Figure 7 shows large-area (a) SHG image and (b) α image of the 10-week-UVB-exposed 16-week-old skin (image size=3.6  mm×3.6  mm, corresponding to 1536  pixels×1536  pixels). Due to imperfect stitching, the marginal effects appear as square block with 600-μm interval in SHG image. However, skin wrinkles appeared as black lines along the horizontal direction in SHG image and α image. More interestingly, the collagen fibers along the wrinkle significantly increased the reddish color or decreased the α value. This result also indicates that the collagen fiber orientation is closely related with the wrinkle direction.

## 5 Conclusion

Polarization-resolved SHG microscopy was applied to pre-wrinkled and wrinkled skins of UVB-exposed mouse model to investigate which came first, the wrinkle or the collagen fiber orientation change. The comparison of α images between UVB-exposed and control skin indicated that the collagen fiber orientation change comes first, then the wrinkle appears; in other words, change of collagen fiber orientation is a trigger of wrinkling in cutaneous photoaging. Thus, the proposed method has potential to be used as not only an *in vivo* evaluator for cutaneous photoaging but also an *in vivo* predictor for skin wrinkling. Furthermore, use of a fiber-coupled, handheld, polarization-resolved SHG microscope^17^ will accelerate its clinical application *in vivo*.
